# Prognostic Analysis of Seven Cases of Sinus Cysts With Visual Dysfunction

**DOI:** 10.7759/cureus.110555

**Published:** 2026-06-09

**Authors:** Yumi Takemiya, Yuichi Teranishi, Kishiko Sunami

**Affiliations:** 1 Department of Otolaryngology - Head and Neck Surgery, Graduate School of Medicine, Osaka Metropolitan University, Osaka, JPN

**Keywords:** diplopia, sinus cysts, visual dysfunction, visual field impairment, visual impairment

## Abstract

Introduction

Sinus cysts associated with visual impairment require surgical drainage of the cyst, and emergency surgery is often necessary, particularly in cases involving visual impairment. In this study, we examine the optimal timing of surgery with respect to visual prognosis, based on our own cases and a review of the literature.

Materials and methods

From April 2020 to March 2025, seven patients with sinus cysts and visual impairment underwent endoscopic surgery at the Department of Otolaryngology at Osaka Metropolitan University. Of these seven cases, three involved visual acuity impairment, three involved visual field impairment, and one involved diplopia.

Results

We conducted a further analysis of the three cases with decreased visual acuity. All cases of visual impairment underwent emergency surgery on the day of the initial consultation. Those with preoperative visual acuity between 0.1 and 1.0 recovered their original visual acuity. Conversely, the case with light perception showed only slight improvement.

Conclusions

Time from onset to treatment and preoperative visual acuity were considered important factors in the postoperative visual prognosis. For patients with visual impairment, especially those with severe preoperative visual acuity, emergency surgery was deemed necessary. In cases of diplopia, there is a high likelihood of improvement even with elective surgery performed in a planned manner after preoperative examinations and surgery scheduling. Because few cases of visual field defects have been reported, further case accumulation is warranted.

## Introduction

Sinus cysts are expansile diseases originating from the paranasal sinuses. Pressure on surrounding structures can cause visual dysfunction, including impaired vision [1-3]. The primary treatment is surgical opening of the cyst. When sinus cysts are accompanied by visual dysfunction, they can affect the prognosis of visual function, so emergency surgery is often required. However, there is currently insufficient evidence to clearly determine the optimal timing of surgery or the prognosis for visual function in sinus cysts associated with visual impairment, and no unified treatment protocol has yet been established.

Therefore, this study aimed to examine the relationship among preoperative visual acuity, the interval from symptom onset to surgery, and postoperative visual outcome in patients with paranasal sinus mucoceles associated with visual disturbance. Additionally, we sought to characterize differences in visual dysfunction, such as diplopia and visual field defects, and to consider the timing of surgery according to the presenting symptoms.

## Materials and methods

Study design

This study was a retrospective case series of seven patients with paranasal sinus cysts associated with visual impairment.

Study participants

Seven patients with paranasal sinus cysts associated with visual dysfunction underwent endoscopic sinus surgery at the Department of Otolaryngology at Osaka Metropolitan University between April 2020 and March 2025. These included three patients with decreased visual acuity, three with visual field defects, and one with diplopia. The inclusion criteria were patients with paranasal sinus cysts associated with visual disturbance who underwent surgical treatment at our department and had sufficient clinical data available for analysis. The exclusion criteria were patients without visual disturbance, those treated conservatively rather than surgically, those with visual symptoms attributable to other causes, and those with insufficient clinical or ophthalmologic data.

Surgical procedure and visual assessment

Endoscopic sinus surgery was performed under general anesthesia in all cases. All procedures were performed by the same surgeon. The cyst was opened to achieve adequate drainage, and the extent of marsupialization was determined according to the lesion location and extent of expansion. Bony decompression of the optic canal was not performed in any case.

Visual function was assessed in collaboration with the department of ophthalmology. Visual acuity was measured using standard ophthalmologic examinations, and postoperative visual acuity was evaluated at one week after surgery. Visual field defects were assessed based on Goldmann perimetry. Diplopia was determined according to the patient’s subjective symptoms and ocular motility findings. Visual disturbances were classified as decreased visual acuity, visual field defects, and diplopia.

Data collection

Clinical data collected from the medical records included patient demographics, medical history, lesion location, preoperative visual acuity, interval from symptom onset to surgery, and postoperative changes in visual function. In this study, emergency surgery on the day of the initial visit was defined as surgery performed on the same day as the first presentation. Symptom onset was determined by the date of visual symptom onset reported by the patient, and the interval from symptom onset to surgery was calculated as the number of days between symptom onset and surgery.

Statistical analysis

Because of the small number of cases, the analysis was primarily descriptive.

## Results

Of the seven cases of sinus cysts associated with visual impairment, four were male and three were female, with ages ranging from 46 to 80 years (median age 54 years). The primary sites of occurrence were the anterior ethmoid sinus in two cases, the posterior ethmoid sinus in three cases (including one Onodi cell), the frontal sinus in one case, and the sphenoid sinus in one case. Among the seven cases with visual dysfunction, the primary manifestations were decreased visual acuity in three cases, visual field defects in three cases, and diplopia in one case.

Table 1 summarizes the characteristics of the seven cases presenting with visual impairment. Surgery was performed on the day of the initial visit in all three cases of visual impairment. In cases with preoperative visual acuity ranging from 0.1 to 1.0, vision was almost fully restored postoperatively. By contrast, improvement was limited to 0.1 for light perception.

**Table 1:** List of sinus cysts associated with visual impairment LP: light perception, N/A: not applicable

Case	Age	Sex	Part	Symptoms	Days from onset to surgery	Preoperation vision	Postoperative vision	Outcome
1	73	Male	Anterior ethmoid sinus	Diplopia	57	N/A	N/A	Improvement
2	50	Male	Anterior ethmoid sinus	Visual field defects	60	N/A	N/A	Improvement
3	54	Male	Frontal sinus	Visual field defects	43	N/A	N/A	Improvement
4	53	Male	Sphenoid sinus	Visual field defects	6	N/A	N/A	Improvement
5	46	Female	Posterior ethmoid sinus	Visual impairment	14	1.0	1.2	Improvement
6	80	Female	Posterior ethmoid sinus	Visual impairment	3	0.1	0.8	Improvement
7	76	Female	Posterior ethmoid sinus (Onodi cell)	Visual impairment	4	LP	0.1	Improvement

## Discussion

In this study, even when emergency surgery was performed on the same day for paranasal sinus cysts accompanied by visual impairment, poor preoperative vision was associated with a poor visual prognosis. These results suggest that preoperative visual acuity may be related to visual outcomes.

Paranasal sinus cysts are benign conditions that grow slowly but can compress adjacent tissues, such as the orbit or brain, causing various symptoms. Regarding visual impairment, there is a report that visual dysfunction was observed in 93 of 457 cases (20.35%) of paranasal sinus mucous cysts [4], while in our series, three patients presented with visual impairment, representing 14.3% of all 21 patients with paranasal sinus cysts who visited our hospital and underwent surgery under general anesthesia during the same period. It is considered that the site of cyst development and the extent of lesion progression are involved in visual impairment associated with paranasal sinus cysts [1,4-6]. Cysts arising in the posterior paranasal sinuses, specifically the sphenoid sinus, the posterior ethmoid sinus, and particularly the Onodi cell, are located close to the optic nerve and are therefore at higher risk for optic nerve dysfunction [1,2,6,7]. Additionally, as cysts enlarge, they can extend into the orbit, nasopharynx, or intracranially; in particular, orbital extension may contribute to visual impairment by affecting the optic nerve or orbital contents [4]. Similarly, in our cases, all patients who experienced visual impairment had cysts that primarily originated in the posterior ethmoid sinus, and one of these was located in the Onodi cell.

On the other hand, the time from symptom onset to treatment and preoperative visual acuity appear to be important factors. Morita et al. reported that cases in which surgery was performed within one month of onset and where preoperative visual acuity loss was mild had the potential for visual improvement. In contrast, cases with prolonged visual impairment showed little improvement [8]. Furthermore, Zukin et al. found in their study of 207 cases of paranasal sinus cysts with visual impairment that postoperative visual recovery was favorable when surgery was performed within six days of onset, and preoperative visual acuity was at least hand motion or better [9]. Based on these reports, preoperative visual acuity and the time until surgery are important factors in improving postoperative visual acuity.

Additionally, Yoshida et al., in their study of 21 cases of paranasal sinus cysts with optic neuropathy, reported a significant positive correlation between preoperative and postoperative visual acuity [7]. Also in our study, surgeries were performed on the day of the initial consultation for three cases with preoperative visual acuity of light perception, 0.1, and 1.0. While visual acuity recovered in the cases with preoperative values of 0.1 and 1.0, there was only minimal improvement in the case with light perception. These findings suggest that visual recovery may be limited in patients with severe preoperative visual impairment even when surgery is performed early. Therefore, although the usefulness of emergency surgery cannot be ruled out, the possibility of irreversible optic nerve damage should also be considered in cases of advanced severity. Currently, due to work-style reforms for physicians in Japan, there are calls to restrict overtime. However, for patients with paranasal sinus cysts and severe visual impairment, the time from onset to surgery affects prognosis, making emergency surgery at the earliest possible opportunity necessary.

Visual field defects refer to conditions where a part of the visual field is impaired, while visual acuity impairment occurs when the central part of the visual field is affected. Therefore, visual acuity impairment is considered a subset of visual field defects. Within the scope of our search, we did not find any reports that specifically addressed the outcomes of visual field defects. However, in our own cases (Case 2, Case 3, and Case 4), surgery was performed 60, 6, and 47 days after onset, respectively, and improvement of the visual field defects was observed. Because visual impairment may occur as the area of visual field loss expands, relatively early surgical intervention may also be warranted.

Diplopia results from compression by expansive lesions, which can impair the oculomotor, trochlear, or abducent nerves or the extraocular muscles within the orbit. Previous studies have indicated that improvement in diplopia is significantly greater than that in visual acuity impairment, with a more favorable prognosis [9]. In our own Case 1, 57 days had elapsed from onset to surgery, but diplopia still improved. In cases of sinus cysts presenting with only diplopia, it is suggested that elective surgery, rather than emergency surgery, may be an option.

This study has several limitations. First, this was a small retrospective study involving seven patients, and only three cases with decreased visual acuity were examined in detail. Therefore, the generalizability of the findings is limited. Second, potential confounding factors, such as coexisting ophthalmologic or neurologic diseases, were not sufficiently evaluated. Third, symptom onset was determined based on the patient's reported timing and may therefore have been subject to recall bias.

Additionally, because this was a retrospective study, selection bias cannot be excluded, and no blinding was performed. Furthermore, the analysis was primarily descriptive. Therefore, the findings of this study should be interpreted with caution, and future prospective studies involving a larger number of cases are needed.

## Conclusions

Preoperative visual acuity may be associated with postoperative visual prognosis in patients with visual dysfunction due to paranasal sinus mucoceles. However, because this was a small retrospective case series, the results should be interpreted as exploratory and hypothesis-generating. Further validation in a larger prospective study is warranted. Additionally, patients presenting with diplopia may show favorable improvement even when elective surgery is performed after appropriate preoperative evaluation and scheduling. In contrast, because only a limited number of cases with visual field defects have been reported, further accumulation of cases is needed.
